# Continuing Professional Development – Medical Imaging

**DOI:** 10.1002/jmrs.596

**Published:** 2022-05-26

Maximise your CPD by reading the following selected article and answer the five questions. Please remember to self‐claim your CPD and retain your supporting evidence. Answers will be available via the QR code and online at www.asmirt.org/news-and-publications/jmrs, as well as published in JMRS – Volume 69, Issue 4 December 2022.

## Medical Imaging – Original Article

### Comparison between radiographers with sonography education working in remote Australia and radiologists' interpretation of ultrasound examinations.

Williams I, Baird M, Schneider M. (2022) *J Med Radiat Sci*. https://doi.org/10.1002/jmrs.576

1. The diagnostic agreement scoring criteria describes Category 2 as:
  - a. Major discrepancy unlikely to lead to an adverse outcome for the patient
  - b. Major discrepancy that would lead to adverse outcomes for the patient
  - c. Complete agreement
  - d. Minor discrepancy
2. Which of the following level of agreement was found between the radiographers/sonographers and radiologist's reports in this study?
  - a. Acceptable
  - b. Good
  - c. High
  - d. Low
3. The most common ultrasound examinations undertaken by the radiographers/sonographers were:
  - a. Obstetric
  - b. Abdomen
  - c. Musculoskeletal
  - d. Doppler
4. A clinical indication for paediatric examinations included suspected:
  - a. Bowel obstruction
  - b. Intussusception
  - c. Abdominal mass
  - d. Urinary tract infection
5. Which of the following was identified as a limitation of this study?
  - a. This study involved only two remote primary healthcare centres, hence results from this study should not be generalised to metropolitan regions.
  - b. The sample size was too small to produce accurate results, and more cases are required to increase the statistical power.
  - c. The radiographers/sonographers involved in this study received formal training in definitive ultrasound reporting, hence results from this study should not be generalised to the whole profession.
  - d. The range of ultrasound examinations was not varied enough to produce accurate results, hence more anatomical sites should be included in future studies.

**Recommended further reading:**

1. Hardy M, Snaith B, Scally A. The impact of immediate reporting on interpretive discrepancies and patient referral pathways within the emergency department: a randomised controlled trial. *Br J Radiol* 2013; 86(1021): 20120112. https://www.birpublications.org/doi/10.1259/bjr.20120112.
2. Kelly BS, Rainford LA, Grey J, McEntee MF. Collaboration between radiological technologists (radiographers) and junior doctors during image interpretation improves accuracy of diagnostic decisions. *Radiography* 2012; 18(2): 90–95: https://doi.org/10.1016/j.radi.2011.06.002.

## Answers


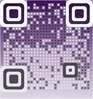


Scan this QR code to find the answers, or visit www.asmirt.org/news-and-publications/jmrs

